# A Case Series Study of Odontoid Fracture in the Elderly: A Severe Fracture Occurring Most Frequently in Osteoporotic Subjects

**DOI:** 10.1002/jbm4.10076

**Published:** 2019-01-10

**Authors:** Lauren Natella, Nicolas Bronsard, Jeremy Allia, Laurent Hekayem, Liana Euller‐Ziegler, Fernand De Peretti, Véronique Breuil

**Affiliations:** ^1^ Rheumatology Universite Cote d'Azur Centre Hospitalier Universitaire (CHU) de Nice Nice France; ^2^ Orthopaedic Trauma and Spine Surgery Universite Cote d'Azur Institut Universitaire de l'Appareil Locomoteur et du Sport Nice France; ^3^ Commission for Atomic Energy and Alternative Energies (CEA)/Directorate of Basic Research (DRF)/Biosciences and Biotechnologies Institute of Aix‐Marseille (BIAM) Unités Mixtes de Recherche (UMR) E4320 Transporters Imaging and Radiotherapy in Oncology (TIRO)–Mécanismes biologiques des Altérations du Tissu Osseux (MATOs) Nice France

**Keywords:** AGING, DXA—ANALYSIS/QUANTITATION OF BONE, CLINICAL TRIALS, OSTEOPOROSIS—DISEASES AND DISORDERS OF/RELATED TO BONE

## Abstract

The WHO definition of osteoporosis excludes cervical fractures. Recent studies suggest that atraumatic odontoid fractures (OF) may be favored by osteoporosis but global bone status for osteoporosis diagnosis has not been described. We present a case series of patients >65 years old hospitalized for low‐energy OF who had an evaluation of their bone status within 3 months after fracture, including clinical risk factors of osteoporosis, bone mineral density (BMD), vertebral fracture assessment (VFA) by dual X‐ray absorptiometry, and laboratory tests. Osteoporosis was defined as a *T*‐score ≤ −2.5 on at least one site, or a bone fragility fracture associated with a *T*‐score ≤ −1 at one site. Thirty‐three patients were hospitalized for OF, 30 of them as a consequence of a low‐energy impact: 20 women and 10 men (mean age: 85 years). Eight patients died before bone evaluation, four refused, and six were lost to follow‐up. Twelve patients were included: 11 women and one man (mean age: 83.8 years). Ten out of twelve patients fulfilled diagnostic criteria of osteoporosis, including eight with previous osteoporotic fractures (six severe fractures). Eight fulfilled specific treatment of osteoporosis criteria, but only two were treated. The mean follow‐up period was 12.2 ± 4.1 months. Prior to OF occurrence, all lived at home and were independent; at the time of discharge, six went to a nursing home. At 3 months of follow‐up (*n* = 10), one was dead and nine lived at home. At 12 months (*n* = 9), two were dead and seven lived at home. This study provides for the first time a classical evaluation of osteoporotic status for low‐energy OF in the elderly and shows that it occurs in osteoporotic subjects. These preliminary results require larger‐scale studies to determine whether OF could be considered as a severe osteoporotic fracture. © 2018 The Authors. *JBMR Plus* is published by Wiley Periodicals, Inc. on behalf of the American Society for Bone and Mineral Research.

## Introduction

The WHO definition of osteoporosis excludes cervical spine fractures.^1^ Epidemiological data report a decrease of cervical diseases incidence for people younger than 65 years old, whereas this incidence remains stable or increases in subjects over 65 years old.^2^, ^3^ Odontoid fracture (OF) is the most frequent fracture of the cervical spine in elderly, and its occurrence is fairly common after low‐energy trauma.^4^, ^5^ OF are severe, and responsible for a high mortality rate.^5^, ^6^, ^7^, ^8^ Given the actual aging trend of the population, an increase in the incidence of OF is expected based on these data and will require the development of appropriate management.^9^, ^10^

Recently, a link between OF in the elderly secondary to a low‐energy trauma and localized osteoporosis of the spine (cervical spine or lumbar spine [L_1_ vertebra]) has been suggested, based on tomodensitometry (CT scan) measurements.^11^, ^12^, ^13^ Watanabe and colleagues^11^ demonstrated by cervical CT scan that osteoarthritis of the C_1_‐C_2_ joint and demineralization of the odontoid were factors predisposing to OF secondary to low‐energy trauma. Emohare and colleagues^12^ found that 65% of patients over 65 years old with OF had osteoporosis measured on the first vertebra by quantitative posterior abdominal CT scan. Kaesmacher and colleagues,^13^ have shown that osteoporosis of the cervical spine measured by quantitative CT scan was the only predictor of OF. However, the diagnosis of osteoporosis according to standard criteria (dual X‐ray absorptiometry [DXA] and risk factors) has never been performed in patients with a low‐energy OF.

This case series describes the overall bone status of patients over 65 years old with a low‐energy OF, according to the standard criteria for osteoporosis diagnosis.

## Patients and Methods

We recorded a case series of hospitalized patients at the Nice University Hospital for OF from January 2016 to April 2017. This study was carried out in compliance with French laws. Informed and written consent was obtained from each patient. The study was recorded on ClinicalTrials.gov (NCT02800278).

The inclusion criteria were: age ≥65 years old, hospitalized for a low‐energy OF. The exclusion criteria were as follows: age <65 years old, OF secondary to a high‐energy trauma, spine metastasis or primary spine tumors, and refusal of bone status evaluation. The OF was diagnosed from standard X‐ray and/or CT scan and/or MRI.

Bone status was evaluated from 1 to 3 months after the fracture event, and included the following:

- Demographic data
- Osteoporosis risk factors: personal history of osteoporotic fractures, parental history of hip fracture, steroid therapy (current or past >7.5 mg/day for >3 months), smoking, alcohol (>3 units/day), early menopause (<40 years), anticonvulsant therapy, gonadotropin‐releasing hormone (GnRH) analogue treatment or anti‐aromatase therapy, unbalanced hyperthyroidism, rheumatoid arthritis, body mass index (BMI) <19 kg/m^2^.
- Biological analysis to investigate the causes of secondary osteoporosis: blood count, serum creatinine, transaminases, alkaline phosphatase, gamma‐glutamyl transferases, albuminemia, serum calcium, phosphatemia, PTH, 25‐OH vitamin D, serum protein electrophoresis, thyroid‐stimulating hormone (TSH).
- Lumbar spine and femoral neck bone mineral density (BMD) by DXA on a HOLOGIC^®^ QDR 4500 machine (Discovery W model). If DXA measurement turned out to be impossible at the femoral site, BMD was performed at the distal radius.
- Vertebral fracture assessment (VFA) by DXA.

Patients were considered as osteoporotic if they had a *T*‐score ≤ −2.5 standard deviation (SD) on at least one site or a history of bone fragility fracture associated with a *T*‐score < −1 SD on at least one site. In the absence of severe osteoporotic fracture, the Fracture Risk Assessment Tool (FRAX) score was calculated to determine the indication for an anti‐osteoporotic treatment based on French guidelines.^14^, ^15^

The clinical evolution (survival and autonomy) was assessed by a medical consultation or a phone call.

## Results

### Demographic data and osteoporosis risk factors

**Figure 1:**
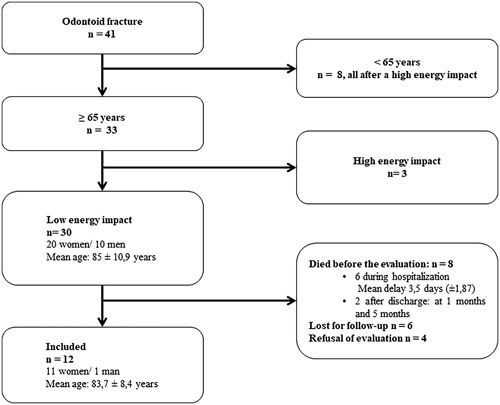
Attrition of the patients hospitalized for odontoid fracture from January 2016 to April 2017 and evaluated for bone status.

A total of 41 patients were hospitalized for OF during the observation period, among which 12 were fully evaluated according our criteria as shown in Fig. 1. Demographic data, osteoporosis risk factors, and osteoporosis treatment history of these 12 patients evaluated fully are summarized in Table 1. All patients had cervical osteoarthritis.

**Table 1:** Demographic Data, Osteoporosis Risk Factors, and Antiosteoporotic Treatment of 12 Subjects With a Low‐Energy Odontoid Fracture

Characteristic	Value
Age (years), mean ± SD	83.75 ± 8.39
Subjects (female/male), *n*	11/1
BMI (kg/m^2^), mean ± SD	24.06 ± 2.40
<19 kg/m^2^	0
Dementia, *n*	1
Previous osteoporotic fracture, *n*	7
Hip fracture in family, *n*	2
Undetermined, *n*	1
Steroid therapy (past or present) >7.5 mg/day, >3 month, *n*	2
Smoking, *n*	6
Weaned, *n*	6
Pack‐years, mean ± SD	6.08 ± 9.12
Alcohol >3 units/day, *n*	2
Early menopause, *n*	1
Aromatase inhibitor, *n*	1
Calcium intake (mg/day), mean ± SD	671.2 ± 219.7
>1000 mg/day, *n*	4
Vitamin and calcium supplementation (*n*)	4
Bisphosphonate, *n*	2
Current, *n*	2
Hormonal replacement therapy, *n*	3

### Biological analysis

The biological evaluation was carried out for 11 of 12 patients. No secondary osteoporosis was discovered. The mean dosage of 25‐OH vitamin D was 30.22 ± 16.67 ng/mL: five patients displayed values <30 ng/mL, including two lower than 10 ng/mL.

### Bone status

Bone status is summarized in Table 2. Femoral BMD could not be achieved in two patients: bilateral total hip prostheses (one with bilateral aseptic osteonecrosis of the femoral heads and one with osteoporotic fractures of the two femoral necks). The VFA could not be performed in one patient due to difficult position maintenance. Five patients had a vertebral fracture, including one discovered in a patient with no fracture history.

**Table 2:** Bone Status of Subjects ≥65 Years Old With a Low‐Energy Odontoid Fracture (*n* = 12)

*T*‐score lowest on measured sites	No fracture (*n*)	Previous osteoporotic fracture (*n*)
>–1 SD	1 patient (male)	0 patients
–1 SD > *T* score > –2.5 SD	1 patient	4 patients
	FRAX 16% (87 years)	1 patient: proximal humeral fracture, wrist fracture, and one vertebral fracture on VFA
		1 patient: two femoral neck fracture and one vertebral fracture on VFA
		1 patient: 5 rib fractures, scapula fracture
		1 patient: right and left wrist fractures; FRAX 41% (83 years) (treatment by biphosphonate)
*T* score ≤ –2.5 SD	2 patients	4 patients
	1 patient: FRAX 17% (89 years)	1 patient: pelvic fracture and left proximal humeral fracture and vertebral fracture on VFA
	1 patient: *T* score ≤ –3 SD	1 patient: vertebral fracture on VFA
		1 patient: right proximal humeral fracture, 5 rib fractures, right femoral neck fracture, and vertebral fracture on VFA
		1 patient: right and left wrist fractures

Ten of 12 patients were osteoporotic, eight with previous osteoporotic fracture, including six severe osteoporotic fractures (Table 2).^14^ According to current French guidelines, eight patients met the criteria for specific osteoporosis treatment.^15^

### Evolution

Five patients required surgery. The mean hospital stay was 11.1 ± 4.7 days (range, 4 to 21 days) and the mean follow‐up period was 12.2 ± 4.1 months (range, 3 to 17 months). One patient had neurological complications concomitant with the fracture but displayed a total recovery. Three patients had complications during hospitalization: cardiorespiratory arrest, occlusive syndrome, and diabetic imbalance. Before hospitalization, the 12 patients were autonomous and lived at home. At time of discharge, six patients went to a nursing home and six returned at home. At 3 months, one patient was dead, one was still in a nursing home, one was institutionalized, and nine were at home. At 12 months, the follow‐up was available for nine of 12 patients: two patients were dead and seven lived at home.

## Discussion

This case series shows that OF in patients over 65 years old mainly occurs in osteoporotic subjects according to the standard criteria for osteoporosis diagnosis.

According to the literature, we found that 90% of all OF in patients ≥65 years old occurred after a low‐energy trauma whereas all OF in younger patients (<65 years old) were secondary to a high‐energy trauma.^4^, ^5^, ^16^ According to previous studies, we observed cervical spine osteoarthritis in patients with atraumatic OF.^11^, ^12^, ^13^

If CT scan is considered as the gold standard to measure BMD, in common practice DXA remains the reference technique for the diagnosis and management of osteoporosis.^1^ We show that most elderly patients with OF have a low BMD assessed by DXA: 11 of 12 had a *T*‐score < −1 SD, including six with a *T*‐score ≤ −2.5 SD. Although it is difficult to directly compare our results with previous studies in which bone demineralization had been assessed by CT scan and in nonconventional sites of osteoporosis, our results are consistent with these studies.^11^, ^12^ Indeed, using CT scan, Watanabe and colleagues^11^ found severe osteoporosis at the cervical spine in seven of 17 patients with OF, compared to one of 21 in the control group; on the first lumbar vertebra, Emohare and colleagues^12^ found osteoporosis in 65% of patients; and Kaesmacher and colleagues^13^ reported osteoporosis at the cervical spine in 53 of 92 patients with OF, and 98 of 296 in the control group.

Our population displays the classical risk factors for osteoporosis, but the small size of the sample did not allow comparison to epidemiological data. Without considering the OF and according to recommendations for osteoporosis treatments, eight of 12 of our patients required anti‐osteoporotic treatment but only two were treated, consistent with the current deficit of osteoporosis treatment.^15^, ^17^

According to the literature in which OF is recognized as severe with an increased mortality (25% at 1 month, 34.4% at 6 months, and 37.5% at 12 months), we found 20% of early mortality (within 7 days), 23.3% at 1 month, 26.6% at 3 months, and 33.3% at 12 months.^6^ This severity is close to what can be observed after osteoporotic femoral neck fracture: 15% to 25% at 1 year.^14^ This is in agreement with Venkatesan and colleagues,^6^ who compared the mortality after OF at 1 year (37.5%) to an age‐matched group of patients with osteoporotic femoral neck fractures (32%). Despite this high mortality, neurological complications in patients with OF are rare: only one patient in our case series, similar to what has been reported in Lefranc and colleagues^18^ (1/27 patients). In our case series, the severity of this fracture is also related to a loss of autonomy: after discharge from the hospital, 50% of patients required a nursing home and one was institutionalized. Regarding the economic costs induced by the OF, the average length of stay was 11.1 days, which is slightly less than what has been reported elsewhere: 15 ± 8.6 days or 13.8 days (for surgically treated patients). This figure can be explained by both the small number of patients of our case series and the fact that only five patients had surgery.^19^, ^20^

The major limitations of this work are the small size of the sample and the lack of a control group. The high dropout rate is mainly due to the elevated early mortality related to this fracture (26% of OF). Moreover, the very old age of the population and the severity of the fracture may also explain the lack of follow‐up after 3 months. Although we should have evaluated bone status in elderly patients with high‐energy OF as a control group, we recorded only three high‐energy OF fractures during the same period; in addition, previous studies show that OF in the elderly most commonly occurs after low‐energy trauma.^4^, ^5^ Although we did not include others cervical fractures as a control group, it is worth noting that Kaesmacher and colleagues^13^ showed that other cervical spine fractures had a higher BMD measured by CT scan. Moreover, epidemiological data show that OF is the most frequent cervical fracture in the elderly, which can be explained in part by the modification of the mechanical stresses.^4^, ^5^ Indeed, in the healthy subject, the segment C_4_–C_7_ is the most mobile segment of the cervical spine. Cervical osteoarthritis predominates on the C_4_–C_7_ segment, inducing rigidity and thereby modifying mechanical stresses: the C_1_–C_2_ segment thus becomes the most mobile portion of the cervical spine and the association of the odontoid base demineralization with the atlanto‐axial osteoarthritis results in joint rigidity and promotes fracture.^11^ According to Watanabe and colleagues,^11^ who concluded that the association between atlanto‐odontoid osteoarthritis and osteoporotic dens‐body junction predisposes older people to OF, all of our patients had cervical spine osteoarthritis. However, Kaesmacher and colleagues^13^ have shown that osteoporosis of the cervical spine measured by quantitative CT scan was the only predictor of OF, independently of osteoarthritis.

## Conclusion

This case series provides for the first time a classical evaluation of osteoporotic status for low‐energy OF in the elderly and shows that it occurs in osteoporotic subjects. These preliminary results require larger‐scale studies to determine whether OF could be considered as a severe osteoporotic fracture.

## Disclosures

VB recieved research grants from Amgen, Honoraria from Amgen and Lilly
